# Digital media use and blood pressure in adolescents

**DOI:** 10.1007/s00431-026-06817-6

**Published:** 2026-03-03

**Authors:** Hande Yetişgin, Pervin Demir, Mihriban Inözü, Esra Çöp, Sare Gülfem Özlü

**Affiliations:** 1 Department of General Pediatrics, Ankara Yıldırım Beyazıt University, Ankara City Hospital, Ankara, Turkey; 2 Faculty of Medicine, Department of Biostatistics, Ankara Yıldırım Beyazıt University, Ankara, Turkey; 3 Ankara Yıldırım Beyazıt University, Faculty of Medicine, Department of Pediatric Nephrology, Ankara, Turkey; 4 Department of Child And Adolescent Psychiatry, University of Health Sciences, Ankara Bilkent City Hospital, Ankara, Turkey; 5 Faculty of Medicine, Department of Pediatric Nephrology, Ankara Yıldırım Beyazıt University, Ankara Bilkent City Hospital, Ankara, Turkey

**Keywords:** Adolescent, Ambulatory Blood Pressure Monitoring, Childhood Hypertension, Problematic Internet Use, Video Game

## Abstract

Background: Hypertension is an increasingly important health concern among children and adolescents. Beyond traditional risk factors such as obesity, sedentary behaviors, including prolonged internet use and video gaming, may contribute to elevated blood pressure. This study aimed to investigate the association between problematic internet use, video gaming, and ambulatory blood pressure parameters in adolescents. Methods: This cross-sectional study included adolescents aged 12–18 years who were referred to a pediatric nephrology outpatient clinic for evaluation of hypertension. Demographic, clinical, and laboratory data were obtained from medical records. Ambulatory blood pressure monitoring (ABPM) was performed to classify ambulatory hypertension and white coat hypertension. Internet use and gaming behaviors were assessed using the Young Internet Addiction Scale (YIA-SF) and the Internet Gaming Disorder Scale (IGDS9-SF). Scale scores were compared with ABPM parameters. Results: A total of 107 adolescents (40 girls, 67 boys) with a mean age of 14.9 ± 1.8 years were included. Based on ABPM findings, 55 participants (51.4%) had ambulatory hypertension, while 52 (48.6%) had white coat hypertension. Higher YIA-SF scores were weakly but significantly associated with higher mean daytime systolic blood pressure. IGDS9-SF scores were not significantly associated with ambulatory blood pressure parameters.

*Conclusion*: Problematic internet use may be associated with higher mean daytime systolic blood pressure in adolescents, including among non-obese individuals. Although these behaviors were not independent predictors of ambulatory hypertension after adjustment for demographic and anthropometric factors, awareness of screen-based behaviors may complement lifestyle counseling in adolescents evaluated for elevated blood pressure.

**Table:** 

**What is Known:** • *The majority of pediatric studies examining the relationship between screen-based behaviors and blood pressure have been based on office blood pressure measurements, which may not fully capture circadian blood pressure patterns compared with ambulatory blood pressure monitoring*.	
**What is New:** • *This study contributes to the limited literature examining the association between problematic internet use, video gaming, and ambulatory blood pressure parameters in adolescents*. • *Higher internet addiction scores were associated with higher mean daytime blood pressure, including among non-obese adolescents*.

**What is Known:**

• *The majority of pediatric studies examining the relationship between screen-based behaviors and blood pressure have been based on office blood pressure measurements, which may not fully capture circadian blood pressure patterns compared with ambulatory blood pressure monitoring*.

**What is New:**

• *This study contributes to the limited literature examining the association between problematic internet use, video gaming, and ambulatory blood pressure parameters in adolescents*.

• *Higher internet addiction scores were associated with higher mean daytime blood pressure, including among non-obese adolescents*.

## Introduction

Hypertension is an increasingly important public health problem among children and adolescents, as highlighted in both earlier epidemiological studies and recent clinical guidelines [1, 2].The rising prevalence of pediatric hypertension has been strongly linked to the global childhood obesity epidemic, a relationship consistently reported across different populations and time periods [1–5]. Numerous population-based studies have demonstrated that overweight and obese children have significantly higher blood pressure (BP) levels compared with their normal-weight peers, findings that are supported by both earlier cohort studies and more contemporary pediatric data [6–9].

In addition to excess adiposity, unhealthy lifestyle patterns, including fast food consumption, insufficient sleep, smoking, and sedentary behaviors, have been identified as major contributors [10]. More recently, emerging evidence suggests that excessive screen exposure, prolonged internet use, and video gaming may not only promote obesity by reducing physical activity, but may also be independently associated with elevated BP in youth [3, 9–13]. Although the underlying mechanisms are not fully understood, proposed pathways include sleep disturbance, increased sympathetic nervous system activity, and sustained cognitive arousal during screen-based activities [14, 15].

Importantly, most previous studies have relied on office-based or casual BP measurements, which are less reliable than ambulatory BP monitoring (ABPM) in detecting true hypertension and circadian BP patterns. In this study, we aimed to evaluate the effects of problematic internet use and video gaming on ABPM parameters in adolescents aged 12–18 years. ABPM has significantly improved our understanding of BP patterns, particularly the role of nocturnal hypertension and masked hypertension, in determining disease severity and long-term outcomes. Importantly, evidence from pediatric studies indicates that these associations are not limited to adults. ABPM has revealed clinically relevant BP abnormalities that may not be detected during office measurements. These findings highlight the necessity of ABPM in pediatric populations to accurately assess BP patterns and identify early target organ damage. Data examining the relationship between screen-based behaviors and ABPM findings in pediatric populations remain limited. In light of this gap, the present study was designed to contribute to the existing literature by investigating the association between screen-based behaviors and ABPM parameters in this age group.

## Patients and methods

This cross sectional study was carried out in our pediatric nephrology outpatient clinic between 1 September 2021 and 31 December 2021. During the study period, approximately 260 adolescents aged 12–18 years were referred to the pediatric nephrology outpatient clinic for evaluation of elevated BP and were assessed for eligibility. Patients with known hypertension, chronic kidney disease, chronic cardiac or endocrine disorders, or diagnosed psychiatric diseases were excluded to rule out secondary hypertension. In addition, patients who declined participation or had incomplete ABPM or questionnaire data were excluded. After applying the predefined exclusion criteria, 107 adolescents were included in the final analysis.

Age, sex, family history, anthropometric measurements, body mass index (BMI), and physical examination findings were recorded. BMI values were calculated as kg/m^2^ and classified according to age- and sex-specific BMI z-scores based on World Health Organization (WHO) growth standards. A BMI z-score < −2 SD was defined as underweight, values between −2 and + 1 as normal; > + 1 SD to + 2 SD were classified as *overweight,* and values > + 2 SD were considered *obesity* [16].

## Laboratory investigations and imaging techniques

Urine analysis, renal function tests (serum, blood urea nitrogen, creatinine, glomerular filtration rate according to the Schwartz formula), uric acid, electrolytes, venous blood gas, cholesterol, triglyceride, renin and aldosterone levels, thyroid function tests, urinary ultrasound, and renal artery Doppler ultrasound were performed to detect the underlying etiology. Urine protein excretion, echocardiography, and ophthalmological examination were carried out to identify end-organ damage.

## Blood pressure measurements

### Office BP measurements

Office BP was measured by auscultation with a calibrated sphygmomanometer. Measurements were performed after at least 5 min of rest in the seated position, using an appropriately sized cuff, in accordance with standard pediatric BP measurement guidelines. Hypertension was defined according to the 2017 American Academy of Pediatrics guideline as BP ≥ 95th percentile for age, sex, and height in children < 13 years, or ≥ 130/80 mmHg in children ≥ 13 years [17].

### Ambulatory BP monitoring

All children underwent ambulatory BP monitoring to confirm the diagnosis of hypertension with a “Spacelabs Healthcare On Trak Ambulatory Blood Pressure Monitor”. To avoid artifacts, a nondominant arm was used for monitoring. An appropriate cuff size was selected according to the hypertension guidelines [17, 18]. BP was recorded every twenty minutes during the awake period and every 30 min during the sleep period.

ABPM was performed over a 24-h period that included both daytime and nighttime measurements. ABPM recordings were generally obtained on regular school days, and monitoring during weekends or school holidays was avoided to minimize variability in daily routines and screen exposure patterns. Children were allowed to continue their daily activities during monitoring, including attending school, but heavy exercise, such as sports participation, was abstained. A diary to record wake and sleep periods, daily activities, and any medications during the 24-h period was requested from all participants. Valid measurements above 90% were considered significant. Incorrect and insufficient measurements were excluded from the study.

Overall mean systolic and diastolic BP (SBP/DBP); daytime and nighttime mean SBP and DBP were evaluated. Systolic and diastolic dipping parameters were determined. A decrease of 10% or more in systolic or diastolic BP between daytime and nighttime measurements was defined as dipping [17, 18].

Although the 2022 AHA pediatric ABPM guidelines, hypertension was defined as elevated mean ambulatory BP (≥ 95th percentile in children aged < 13 years or meeting adolescent cut points in those aged ≥ 13 years: 24-h ≥ 125/75 mmHg and/or wake ≥ 130/80 mmHg and/or sleep ≥ 110/65 mmHg). White coat hypertension was defined as elevated office BP with normal ABPM. Ambulatory hypertension was defined as the presence of both elevated office BP and elevated ambulatory BP.

Although the 2022 AHA pediatric ABPM guidelines define four BP phenotypes (normotension, masked hypertension, white coat hypertension, and ambulatory hypertension), our study population consisted exclusively of children referred for elevated office BP. Therefore, masked hypertension and true normotension were not represented by design. So, participants were accordingly classified as having ambulatory hypertension or white coat hypertension (WCH).

Problematic internet use and gaming behaviors were assessed using validated self-report questionnaires with Young Internet Addiction Short Form (YIA-SF) and Internet Gaming Disorder-Scale Short Form (IGDS9-SF**)** as described in the following section, which evaluate habitual patterns of internet and gaming use rather than time-specific behaviors during ABPM.

## Young internet addiction short form (YIA-SF)

To evaluate problematic internet use, the Young Internet Addiction Short Form was administered to all participants.

The YIA-SF consists of twelve questions evaluating the frequency and duration of internet use of the patients and the effect of the internet on daily social life and school life. It is a 5-point Likert-type scale and is scored as 1 = never to 5 = always. There is no reverse-scored item in the scale. The minimum scale score is 12, the total scale score is 60, and higher scores indicate an increased risk of internet addiction [19]. In this study, we used the Turkish version of the test, which was generated and validated by Kutlu et al. [20]. The validation of the test was performed among adolescents and university students.

## Internet gaming disorder-scale short form (IGDS9-SF)

To determine the effects of video gaming, the Internet Gaming Disorder Scale Short Form (IGDS9-SF) was administered to all participants.

This scale was developed by Pontes HM and Griffiths MD, and the first Turkish version was developed and applied by Günüç S and Kayri M in 2017 [21]. In 2018, Evren et al. demonstrated the validity and reliability of this scale among young Turkish adults [22]. The scale consists of nine questions about the time spent on computer game activities and electronic devices such as game consoles, mobile phones, tablets, and all kinds of games that can be played both online and offline. The scale consists of nine items. Each item is rated on a 5-point Likert scale ranging from 1 = never to 5 = very often. The maximum and minimum scores of the IGDS9-SF are 9 and 45, respectively. Higher scores indicate higher levels of problematic internet gaming.

## Ethical issues

Ethical approval was obtained from the Research and Ethics Committee of Ankara Bilkent City Hospital (date: 02.12.2020 no: E2-20–14). Detailed information was given, and written informed consent was obtained from both children and parents who volunteered to participate in this study.

## Statistical analysis

Statistical analyses were performed using SPSS version 25.0 (IBM Corp., Armonk, NY, USA). The normality of continuous variables was assessed using the Kolmogorov–Smirnov and Shapiro–Wilk tests**,** supported by visual inspection of histograms and Q–Q plots**.** Continuous variables were presented as mean ± standard deviation (SD)**,** and categorical variables as frequencies and percentages**.**

Participants were classified as having ambulatory hypertension or white coat hypertension based on ABPM findings. Comparisons between ambulatory hypertension and WCH groups were performed using independent-samples t-tests for continuous variables and χ^2^ tests for categorical variables. A p-value < 0.05 was considered statistically significant.

Behavioral scale scores (YIA-SF and IGDS9-SF) were compared between BP groups using independent-samples t-tests. Associations between scale scores and parameters were evaluated using Pearson correlation analysis.

To identify independent predictors of hypertension, a multivariable binary logistic regression model was constructed with hypertension status as the dependent variable. The model included age, sex, BMI category, Young Internet Addiction Test score, and video game scale score as covariates. Results were reported as adjusted odds ratios (ORs**)** with 95% confidence intervals (CIs). Additionally, multiple linear regression was used to examine determinants of mean daytime systolic BP, with age, sex, BMI category, YIA-SF score, and IGDS9-SF score entered as predictors. Model performance was evaluated using R^2^ and F statistics.

Given the exploratory nature of the analyses, adjustments for multiple comparisons were not applied; therefore, findings should be interpreted cautiously. A two-sided p value < 0.05 was considered statistically significant.

## Results

### Demographic and clinical characteristics

A total of 107 adolescents were included in the study, of whom 40 (37.4%) were girls and 67 (62.6%) were boys, with a mean age of 14.9 ± 1.8 years. Based on BMI classification, 29 (27.1%) were normal weight, 32 (29.9%) overweight, and 46 (43.0%) obese (Table 1).

**Table 1:** Characteristics Of The Study Population According To Blood Pressure Phenotype

Characteristic	Total (n = 107)	WCH (n = 52)	Ambulatory Hypertension(n = 55)
Age (years), mean ± SD	14.93 ± 1.84	14.71 ± 1.84	15.14 ± 1.83
Sex, n (%)			
Male	67 (62.6)	32 (61.5)	35 (63.6)
Female	40 (37.4)	20 (38.5)	20 (36.4)
BMI category, n (%)			
Normal	29 (27.1)	11 (21.2)	18 (32.7)
Overweight	32 (29.9)	18 (34.6)	14 (25.5)
Obesity	46 (43.0)	23 (44.2)	23 (41.8)

BMI: Body Mass Index

WCH: White Coat Hypertension

No secondary causes of hypertension were identified, and all cases were classified as primary hypertension. Left ventricular hypertrophy was detected in 23 hypertensive individuals, while ophthalmologic evaluations were normal in all participants.

### Ambulatory BP categories

According to ABPM results, 55 patients (51.4%) had ambulatory hypertension, while 52 (48.6%) had WCH.

### Relationship of BP categories with age, sex and BMI

When BP categories were compared according to demographic and anthropometric characteristics, age did not differ significantly between patients with WCH and ambulatory hypertension **(**p = 0.234). Similarly, the distribution of sex was comparable across BP categories **(**p = 0.961). BMI categories were also not significantly associated with BP status (p = 0.349) (Table 1).

### Internet use and gaming scores within demographic subgroups

YIA-SF scores were comparable between boys and girls, whereas IGDS9-SF scores tended to be higher in boys. BMI category and weight status did not show clear associations with either YIA-SF or IGDS9-SF scores. A weak inverse relationship was observed between age and YIA-SF scores, while IGDS9-SF scores showed a similar but less consistent pattern.

### Behavioral scores and BP categories

Young Internet Addiction Test scores were similar between the WCH and ambulatory hypertension groups **(**27.10 ± 8.62 vs. 28.38 ± 7.83**,** respectively), with no statistically significant difference (F(1,105) = 0.653, p = 0.421). Likewise, video game scale scores did not differ significantly between the groups (16.33 ± 5.93 vs. 17.16 ± 7.49, t(105) = 0.638, p = 0.525) (Table 2).

**Table 2:** Comparison of Young Internet Addiction Test and Internet Gaming Disorder Scale–Short Form Scores According to ABPM Phenotype

Variable	WCH (n = 52)	Ambulatory Hypertension(n = 55)	p value
YIA-SF (mean ± SD)	27.10 ± 8.62	28.38 ± 7.83	0.421
IGDS9-SF (mean ± SD)	16.33 ± 5.93	17.16 ± 7.49	0.525

Values are presented as mean ± standard deviation (SD). P values were calculated using the independent-samples t test

YIA-SF Young Internet Addiction Short Form

IGDS9-SF:Internet Gaming Disorder Scale Short Form

WCH: White Coat Hypertension

### Correlation between behavioral scores and office BP measurements and ABPM parameters

Office BP measurements showed no significant associations with YIA-SF or IGDS9-SF scores.Higher YIA-SF and IGDS9-SF scores were associated with higher mean systolic BP, although these correlations did not reach statistical significance (YIA-SF: r = 0.168, p = 0.084; IGDS9-SF: r = 0.025, p = 0.80). No significant correlations were found between behavioral scores and diastolic BP (Table 3).

**Table 3:** Correlations between office BP, ABPM parameters, and YIA-SF and IGDS9-SF Scores

	**YIAS-SF**		**IGDS9-SF**	
BP Categories (Office/ABPM)	**r/r**_**s**_	**p**	**r**_**s**_	**p** ^a^
Office systolic BP	−0.026	0.79^a^	−0.124	0.20
Office diastolic BP	0.007	0.94^a^	−0.062	0.53
Heart Rate	0.035	0.72^a^	0.102	0.30
Mean systolic BP	0.168	0,084^a^	0.025	0.80
Mean diastolic BP	0.152	0.12^a^	−0.011	0.91
Daytime systolic BP	0.191	**0.049**^a^	0.027	0.78
Daytime diastolic BP	0.118	0.23^a^	−0.024	0.81
Nighttime systolic BP	0.110	0.26^b^	0.070	0.48
Nighttime diastolic BP	0.136	0.16^a^	0.015	0.88
Systolic dipping	−0.084	0.39^a^	−0.094	0.34
Diastolic dipping	−0.062	0.52^a^	−0.044	0.66

Abbreviations: BP, blood pressure; YIA-SF, Young Internet Addiction Short Form; IGDS9-SF, Internet Gaming Disorder Scale–Short Form. Footnotes: Spearman or Pearson correlation coefficients were used as appropriate. Statistically significant values are shown in bold

^a^:Spearman

^b^:Pearson

YIA-SF scores showed weak but statistically significant correlations with mean daytime BP whereas IGDS9-SF scores were not significantly correlated with any systolic ABPM parameter (Table 3). Nighttime systolic and daytime/nighttime diastolic BP increased with higher YIA-SF scores, though not significantly.

### Subgroup analysis: obese vs. non-obese participants

For subgroup analyses, normal-weight and overweight participants were combined into a single non-obese group to improve statistical power. Among non-obese adolescents, higher YIA-SF scores were significantly associated with higher mean daytime systolic BP (r = 0.280, p = 0.019). This association was not observed among obese participants, suggesting a potential modifying role of BMI status.

### Predictors of ambulatory hypertension: multivariable logistic regression analysis

In the multivariable binary logistic regression model evaluating predictors of ambulatory hypertension (vs white coat hypertension), none of the included variables were independently associated with ambulatory hypertension. Age was not a significant predictor **(**adjusted OR 0.857, 95% CI 0.686–1.072; p = 0.175**).** Sex was also not associated with ambulatory hypertension **(**male vs. female: adjusted OR 0.993, 95% CI 0.426–2.316; p = 0.987). Compared with the normal BMI category, the BMI category was not a significant predictor of ambulatory hypertension (adjusted OR 1.469, 95% CI 0.872–2.473; p = 0.149**).** Similarly, Young Internet Addiction Test score (per 1-point increase: adjusted OR 0.976, 95% CI 0.915–1.042; p = 0.466) and video game scale score (per 1-point increase: adjusted OR 0.989, 95% CI 0.913–1.072; p = 0.796**)** were not significantly associated with ambulatory hypertension (Table 4).

**Table 4:** Multivariable Binary Logistic Regression Analysis For Predictors Of Ambulatory Hypertension (Vs White Coat Hypertension)

Predictor	Adjusted OR	95% CI (lower)	95% CI (upper)	p value
Age (years)	0.857	0.686	1.072	0.175
Sex (male vs female)	0.993	0.426	2.316	0.987
BMI category (reference: normal)	1.469	0.872	2.473	0.149
YIA-SF (per 1-point increase)	0.976	0.915	1.042	0.466
IGDS9-SF (per 1-point increase)	0.989	0.913	1.072	0.796

OR odds ratio, CI confidence interval. Adjusted ORs are from the multivariable model including all listed predictors, with ambulatory hypertension (vs white coat hypertension) as the dependent variable. P values from Wald test

BMI: Body Mass Index. IGDS9-SF:Internet Gaming Disorder Scale Short Form (IGDS9-SF) YIA-SF: Young Internet Addiction Short Form

### Linear regression analyses for systolic BP

A multiple linear regression model was constructed to evaluate factors independently associated with mean daytime systolic BP (Table 5).

**Table 5:** Linear regression model for mean daytime systolic BP

Predictor	B	SE	β	t	p value
Constant	105.426	7.053	—	14.95	< 0.001
Sex (male)	3.405	1.742	0.182	1.95	0.053
Age	0.961	0.461	0.195	2.09	0.040
BMI category	2.069	1.021	0.188	2.03	0.045

Model summary: R = 0.334; R^2^ = 0.112; F(3,103) = 4.319; p = 0.007

The overall model was statistically significant (F = 4.319, p = 0.007; R^2^ = 0.112), explaining approximately 11.2% of the variance in mean daytime systolic BP.

Age (p = 0.040) and BMI category (p = 0.045) were identified as independent predictors of mean daytime systolic BP. Male sex showed borderline significance (p = 0.053). Neither YIA-SF nor IGDS9-SF scores were independently associated with mean daytime systolic BP in the multivariable model.

#### Screen-based behaviors and target organ damage

Among the patients diagnosed with hypertension, 23 had left ventricular hypertrophy. Young Internet Addiction Test and Internet Gaming Disorder Scale scores did not differ significantly between those with and without left ventricular hypertrophy (YIA-SF: p = 0.92; IGDS9-SF: p = 0.97).

## Discussion

In this study, we investigated the relationship between problematic internet use, video gaming behaviors, and ambulatory BP parameters in adolescents. Although both YIA-SF and IGDS9-SF scores showed a trend toward higher systolic BP, these associations did not reach statistical significance in general correlation analyses. However, we found that YIA-SF scores were positively and significantly associated with mean daytime systolic BP Importantly, this relationship persisted among non-obese adolescents, suggesting that problematic internet use may influence systolic BP independently of BMI.

Problematic internet use is increasingly recognized as a growing public health concern among adolescents, with documented associations with psychological, behavioral, and cardiometabolic risk factors [23]. Several studies have highlighted that excessive screen exposure, including prolonged internet use, may contribute to increased BP, obesity, depression, anxiety, and unfavorable lipid profiles [14, 24, 25]. Cassidy-Bushrow et al. demonstrated that among 331 adolescents, time spent on the internet was positively correlated with BP —particularly diastolic pressure—independent of BMI [13]. These findings align with our observation that internet use may exert physiological effects beyond weight-related mechanisms.

The cardiovascular impact of video gaming has also been documented in the literature. Goldfield et al. reported that screen exposure, particularly video game playing, was independently associated with increased BP and adverse lipid parameters in overweight and obese adolescents [26]. Similarly, Wang et al. observed acute increases in both systolic and diastolic BP during video game play in younger boys [12]. In an interesting study by Siervo et al., the authors the effect of violent video games on BP [11]. The authors suggested that violent video games cause a high cardiac load, which is probably related to the activation of the stress response [11]. They found that diastolic BP increased progressively during violent video games, and they stated that ongoing increases in BP may cause significant cardiac problems. Although our study did not evaluate game content, the existing literature emphasizes that video games—especially competitive or violent content—may activate sympathetic nervous system pathways, thereby increasing BP [11].

WCH represents a distinct clinical phenotype characterized by elevated clinic BP but normal ambulatory measurements. In our cohort, digital media–related scores did not differ significantly between ambulatory hypertension and white coat hypertension groups, suggesting that problematic internet use alone may not explain the persistence of ambulatory hypertension among adolescents presenting with elevated office blood pressure. Nevertheless, given prior evidence linking screen exposure to autonomic activation and cardiovascular stress responses, digital behaviors may still represent a potentially modifiable factor within the multifactorial framework of pediatric BP regulation.

As has been demonstrated in many studies to date, a sedentary lifestyle may be associated with obesity and consequently hypertension. Considering this well-known finding, we tried to evaluate the effects of internet addiction and video games on ABPM parameters independent of obesity. As mentioned above, Siervo et al. demonstrated that video games can increase diastolic BP, especially in nonobese young men [11].In the comprehensive study of Gopinath et al. conducted with 2353 school children with a mean age of 12.7 years, it was determined that screen exposure was significantly associated with BP, independent of BMI and other factors [10]. In line with these observations, our subgroup analyses suggested that higher YIA-SF scores were associated with higher ambulatory systolic BP values among non-obese adolescents. Although these findings should be interpreted cautiously, they raise the possibility that sympathetic activation, arousal, or stress responses during internet use may contribute to systolic BP variability independently of adiposity.

Despite these patterns, neither YIA-SF nor IGDS9-SF scores emerged as independent predictors of ambulatory hypertension in the binary logistic regression model. Similarly, in the multivariable linear regression analysis, age and BMI category were identified as independent predictors of mean daytime systolic BP while behavioral scale scores were not independently associated. These findings suggest that although problematic internet use may be related to certain systolic BP parameters, its effects appear to be modest and potentially mediated by demographic or anthropometric factors.

The differential associations observed between YIA-SF and IGDS9-SF scores and systolic BP warrant further consideration. YIA-SF reflects broader patterns of problematic internet use, which may encompass prolonged screen exposure, disrupted sleep, and sedentary behavior—factors that have previously been implicated in BP regulation. In contrast, IGDS9-SF focuses on gaming-specific behaviors and may capture a narrower behavioral domain that does not necessarily translate into sustained physiological effects on BP. In addition, variability in gaming intensity and duration within our cohort may have limited the ability to detect associations with ABPM parameters.

From a clinical perspective, these findings may have implications for the counseling of adolescents and their families. While screen-based behaviors are often discussed primarily in relation to obesity and mental health, our results suggest that problematic internet use may also be relevant to BP regulation, even among non-obese adolescents. Clinicians evaluating adolescents with elevated BP may consider incorporating questions about internet use patterns into routine counseling, alongside established lifestyle recommendations such as physical activity, sleep hygiene, and dietary habits. Importantly, these findings do not imply causality but highlight the potential value of a more holistic approach to digital behaviors in pediatric hypertension counseling.

The use of ABPM represents a major strength of our study, as it provides a comprehensive evaluation of BP over a 24-h period. In addition to mean BP values, ABPM enables assessment of daytime and nighttime BP patterns and circadian variation, which cannot be captured reliably by office measurements. Therefore, ABPM offers a more accurate and clinically relevant characterization of BP status in our cohort.

## Limitations

The primary limitation of this study is the relatively small sample size, which may have reduced the statistical power to detect weaker associations. Additionally, ABPM was generally performed on weekdays; however, the timing was not strictly standardized for all participants, which may have introduced some variability in daily screen exposure.

Our study population was disproportionately male and included a high prevalence of overweight/obesity. Although these features may reflect real-world clinical practice and the epidemiology of adolescent hypertension, they may also restrict external validity and limit extrapolation of our findings to cohorts with a more balanced sex distribution or lower BMI burden.

Although habitual internet and gaming behaviors were assessed using standardized questionnaires, gaming or screen use was not recorded in a time-linked manner during ABPM. Therefore, the acute effects of gaming on individual BP readings could not be evaluated.

Another limitation is the relatively high proportion of overweight and obese adolescents in the study cohort. As participants were recruited from a tertiary care setting for evaluation of elevated BP this distribution may not reflect the BMI profile of the general pediatric population, potentially limiting the generalizability of our findings.

Another important limitation is the absence of a true normotensive control group. As all participants were referred for evaluation of elevated office BP, comparisons could only be made between adolescents with ambulatory hypertension and those with white coat hypertension. Therefore, our findings cannot be generalized to adolescents with normal BP in the community, and the observed associations should be interpreted within this specific clinical population.

Furthermore, reliance on self-reported questionnaires may have introduced reporting bias, as adolescents may underreport or overreport their internet use and gaming behaviors. Finally, although several potential confounders—such as sleep duration, physical activity, and caffeine intake—were acknowledged in the Introduction, these factors were not directly measured and therefore could not be systematically accounted for in the analyses.

## Conclusion

In this study, we observed that problematic internet use and video gaming behaviors were associated with certain systolic ABPM parameters in adolescents. Although these behaviors were not independent predictors of BP category after adjustment for age, sex, and BMI, higher YIA-SF scores were correlated with higher mean BP particularly among non-obese adolescents. These findings suggest that internet-related behavioral patterns may be relevant to systolic BP regulation through pathways not solely explained by adiposity.

From a clinical perspective, awareness of screen-based behaviors may complement existing lifestyle counseling in adolescents evaluated for elevated BP. Consideration of internet use patterns may be helpful alongside established recommendations regarding physical activity, sleep, and diet. Importantly, these findings do not imply causality but highlight the potential value of a more comprehensive assessment of digital behaviors in this population.

Further studies with larger sample sizes and longitudinal designs are needed to clarify the temporal and causal relationships between internet use, gaming behaviors, and cardiovascular risk in adolescents.

## Data Availability

No associated data.
